# Inhibitory effect of endostatin gene therapy combined with phosphorus-32 colloid on tumour growth in Wistar rats

**DOI:** 10.1042/BSR20160117

**Published:** 2016-06-30

**Authors:** Huiqi Gao, Jing Zhu, Yong Li, Peng Fu, Baozhong Shen

**Affiliations:** * Department of Nuclear Medicine, The First Affiliated Hospital of Harbin Medical University, China; † Department of Gerontology, The First Affiliated Hospital of Harbin Medical University, China; ‡ Department of PET-CT, The First Affiliated Hospital of Harbin Medical University, China; § Department of Radiology, The Fourth Hospital of Harbin Medical University, China; ║ Molecular Imaging Research Center of Harbin Medical University, China

**Keywords:** endostatin, gene therapy, radiotherapy

## Abstract

Eighty healthy male Wistar rats, aged 5 weeks, weighing 100–120 g, were utilized for establishing tumour-bearing models by immediate Walker-256 cancerous ascites injection and randomly divided to four groups (*n*=20) treated with 0.2 ml solution containing saline, ^32^P-colloid (0.3 mCi), endostatin gene (20 μg), endostatin gene combined with colloid ^32^P. The effect of endostatin combined with a small dose of ^32^P-colloidal on tumour growth *in vivo* was evaluated and the potential mechanism underlying the combined therapy was explored. We found that ^32^P-colloid combined with endostatin exhibited higher inhibitory effect upon tumour growth compared with application of ^32^P-colloid or endostatin alone, although three therapies all significantly inhibited tumour growth compared with saline control group. The higher inhibitory effect of ^32^P-colloid combined with endostatin upon tumour growth might be attributed to a synergistic effect of inhibiting angiogenesis by endostatin and inducing apoptosis by ^32^P-colloid, as demonstrated by microvessel density (MVD) and apoptotic index (AI) measurement. Combined therapy of ^32^P-colloid and endostatin probably serves as a novel and efficacious therapy of tumour growth.

## INTRODUCTION

Conventional treatment modalities of cancer, such as surgery, radiotherapy and chemotherapy, have achieved encouraging clinical efficacy for certain tumour types. However, the range of clinical application is still highly restricted due to severe side effects or alternative unfavourable factors. How to alleviate the damages to normal tissues during cancer treatment is to be urgently resolved. Recently, internal radiation therapy with radiopharmaceuticals has gradually captivated widespread attention due to multiple advantages, such as focused and high efficient therapy, small radiation dose and minor damage to adjacent tissues [1]. Folkman in 1970s proposed the concept of angiogenesis-dependent tumour growth [2] and later identified endostatin as an angiogenesis inhibitor produced by hemangioendothelioma [3]. Since then, cancer treatment targeting angiogenesis, especially endostatin gene and its product as an angiogenesis inhibitor, has become a hot research topic [4,5]. Endostatin combined with radiotherapy or chemotherapy has achieved favourable results in experimental research [6–9]. However, the effect of endostatin combined with internal radiation therapy on tumour growth has been rarely reported. In the present study, the effect of endostatin combined with a small dose of ^32^P-colloidal on *in vivo* tumour growth was assessed and the potential mechanism was unravelled.

## MATERIALS AND METHODS

### Reagents

The secreted endostatin plasmid (pCMV-ssEndostatin) was a gift from the Key Laboratory of Radiobiology, Ministry of Health, School of Public Health, Jilin University, China. Lipofectamine 2000 was purchased from Invitrogen. The rabbit antibody against mouse VIII factor related antigen was purchased from Fuzhou Maixin Biotech. Co. The TUNEL kit was purchased from Wuhan Boster Company. ^32^P radioactive colloid with particle diameter 20–50 nm and radiochemical purity of 99% was purchased from Beijing Atomic Hi-Tech Co.

### Tumour cell line

The rat breast cancer cell line Walker-256 was provided by Institute of *Materia Medica*, Chinese Academy of Medical Sciences, China.

### Animals and treatments

Healthy male Wistar rats, 5-week old, weighing 100–120 g, were provided by the Experimental Animal Center at the First Affiliated Hospital of Harbin Medical University. Under sterile conditions, 0.3–0.5 ml of Walker-256 cancerous ascites (approximately 10^5^–10^6^ cells) was injected subcutaneously into the outer side of the right hind root of rat. At 7 days after inoculation, the longest diameter (*L*) and maximum vertical diameter (*W*) of tumours were measured with callipers and the tumour volume was calculated by *V* (mm^3^)=*L* × *W*^2^/2. The rats with similar tumour volume were randomly divided to four groups (*n*=20) treated with 0.2 ml solution containing saline, ^32^P-colloid (0.3 mCi), endostatin gene (20 μg) and endostatin gene combined with colloid ^32^P respectively. The tumour volume was measured at the indicated time after treatment and the tumour growth rate (*f*) was calculated as the ratio of tumour volume after treatment (*V*_t_) and tumour volume before treatment (*V*_0_): *f*=*V*_t_/*V*_0_.

### Western blot

Walker-256 tumour cells were inoculated into the six-well plate at a cell density of 3 × 10^5^ per well. At approximately 90% of cell confluence, Walker-256 cell lines were transfected with 5 μl PBS, 5 μg empty plasmids and 5 μg pCMV-endostatin plasmids respectively. After 36-h transfection, the supernatants were collected for quantitatively measuring the expression levels of endostatin protein by Western blot analysis.

### RT-PCR

For a total of 30 cycles, each PCT cycle consisted of 94°C for 55 s, 58°C for 45 s and 72°C for 30 s for endostatin amplification: 94°C for 55 s, 58°C for 45 s and 72°C for 40 s for GADPH amplification. The PCR products were separated by gel electrophoresis, stained, visualized and photographed. Forward primer for endostatin: TGCCCAGCTCCTGGCCCGCCGCTT, and reverse prime: GTGCATCAACACAGGCGCCTCTTC.

### Tumour morphology observation by light microscopy

All rats were killed at D20 after corresponding treatment. Tumour tissues were fixed in 10% formalin and embedded in paraffin. Tumour slice was stained with haematoxylin and eosin (HE) and tumour morphology was observed under light microscopy.

### VEGF positive rate

Under immunochemical observation, the tumour cells with positive VEGF were stained as yellow and brown cytoplasm. Five fields with high expression of endostatin were selected. Subsequently, the ratio of positive-VEGF cells/total cells was calculated. The average value of the five visual fields was obtained as the final positive rate of VEGF. Immunochemical staining of the tumour cells with positive VEGF was subsequently performed to confirm the accuracy of the calculated outcomes.

### Tumour microvessel density (MVD) measurement

One representative section of tumour from each rat was stained for factor VIII-related antigen using a standard immunohistochemistry technique. The vascular distribution in the whole slice was first observed at 40 times magnification and five regions with the most intensive microvascular distribution, namely ‘hot spots’, were chosen and the number of vessels in each region was counted at 200 times magnification. The average counting of the five regions was indicated as microvessel density (MVD). Endothelial cells or endothelial cell clusters which were brown stained, vessel lumen and erythrocytes were not judged as microvessel.

### Apoptotic Index (AI) measurement

TUNNEL assay was used to measure the Apoptotic Index (AI). Briefly, the paraffin-embedded tissue sections were deparaffinized and dehydrated. A portion of 50 μl of TUNEL reaction mixture was added to tissue sections. Then, on each slide, 50 μl of conversion agent (POD) and 50–100 μl of substrate solution were added successively and slides were incubated for 10 min at room temperature. Slides were then dehydrated, cleared and mounted for co-focal microscopy observation. Positive signal was defined as the presence of a distinct fluorescence staining within the nuclei. AI was determined by counting a total of at least 1000 nuclei subdivided in 20 fields chosen randomly at 400 times magnification. Apoptotic cells were identified by TUNEL in conjunction with characteristic morphological changes, such as cell shrinkage, membrane blebbing and chromatin condensation, to distinguish apoptotic cells and apoptotic bodies from necrotic cells, which were not considered as apoptotic cells. AI was calculated by dividing the number of apoptotic cells by the total number of counted cells in each slide.

### Statistical analysis

All quantification data were expressed as *x*±*S* (mean ±standard deviation). SNK-q test was used for statistical analysis of tumour growth rate, tumour MVD and AI. SPSS13.0 statistical software was used for all data analysis (SPSS).

## RESULTS

### General observation

In the saline control group, the tumours were obviously enlarged with a magenta colour. At late stage, the rats exhibited extremely poor status with sparse hair and thin epidermis. Tumours on these rats were not movable and ulcerations were noted on tumour surface showing white fish flesh-like tissues. Most rats died within 14 days after treatment. In the ^32^P-colloid or endostatin alone group, the animals were generally in good condition without abnormalities in hair and body colour, and presented with slow tumour growth rate and high mobility. Interestingly, the tumours in the ^32^P-colloid combined with endostatin group were unpalpable and flaky and significantly reduced in volume, and some tumours formed scabs and fell off at late stage.

### Western blot

**Figure 1:**
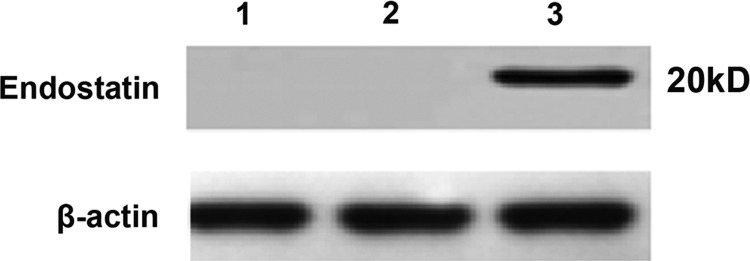
Western blot of expression level of endostatin protein Control (lane 1), empty plasmid (lane 2) and pCMV-endostatin group (lane 3).

The expression level of endostatin protein was detected by Western blot. The results revealed that the expression level of endostatin in the p-CMV-endostatin group (lane 3) was significantly up-regulated compared with those in the PBS (lane 1) and empty plasmid groups (lane 2), as illustrated in Figure 1.

### RT-PCR

**Figure 2:**
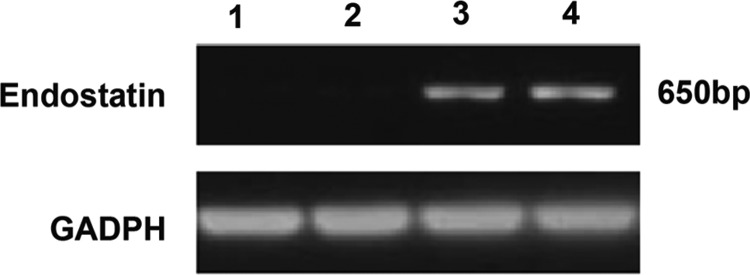
RT-PCT of expression level of endostatin mRNA Control (lane 1), 32P (lane 2), endostatin gene (lane 3) and ^32^P+endostatin gene group (lane 4).

The expression levels of endostatin mRNA among different groups were quantitatively measured by RT-PCR. The results revealed that the expression levels of endostatin mRNA in the endostatin gene (lane 3) and 32P+endostatin groups (lane 4) were significantly up-regulated compared with those in the control (lane 1) and 32P groups (lane 2), as shown in Figure 2.

### Positive rate of VEGF

**Figure 3:**
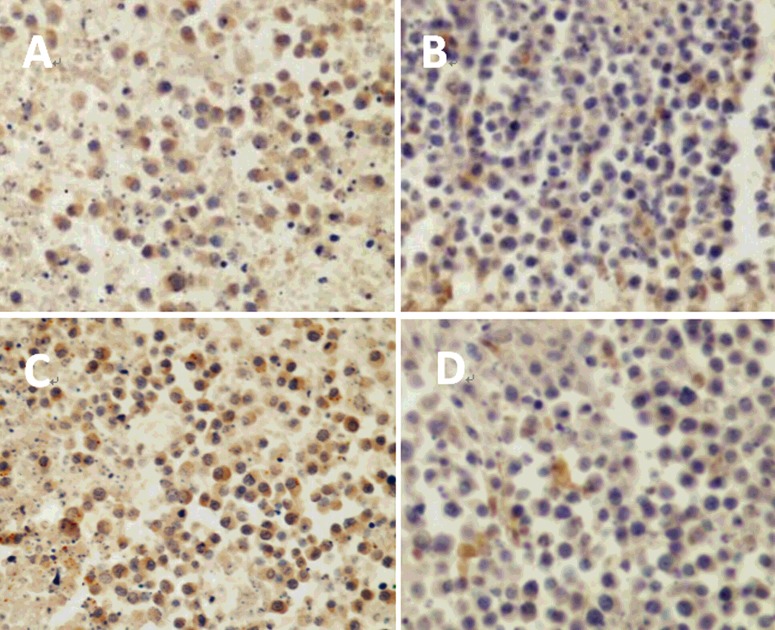
Immunochemical staining of the tumour cells with positive VEGF (×200) Control (**A**), endostatin gene (**B**), ^32^P (**C**) and ^32^P+endostatin gene group (**D**).

The results demonstrated that the positive rate of VEGF in the ^32^P group was (70.08±15.53)%, which was the highest among all groups, followed by (57.68±13.58)% in the control group, (35.46±12.45)% in the endostatin gene group and (18.60±8.62)% in the ^32^P+endostatin group, which was the lowest among four groups. The results of VEGF positive rate were consistent with immunohistochemical staining findings (Figure 3). The tumour cells with positive VEGF were stained as yellow and brown. The quantity of the positive tumour cells in the ^32^P group was the largest among four groups.

### Tumour growth measurements

The tumour growth rates at D7 and D14 after treatment were shown in Table 1. The tumour growth rates in all three treatment groups were significantly lower than that in the saline control group (*P*<0.001). There was no statistical significance in tumour growth rate between the ^32^P-colloid and endostatin gene groups (*P*>0.05). However, the tumours in the ^32^P-colloid combined with endostatin gene group grew at a slower rate compared with either ^32^P-colloid or endostatin gene group (*P*<0.01).

**Table 1:** Comparison of tumour growth rates at 7 and 14 days after treatment among four groups (*x*±*S*) *Note*: *P<0.001 compared with saline control group; ^†^*P*<0.01 compared with ^32^P-colloid or endostatin group.

	Tumour growth rate (*f*=*V*_t_/*V*_0_)	
Group	D7	D14
Saline control	10.52±2.04	17.63±3.62
^32^P-colloid	4.31±1.78*	7.17±2.98*
Endostatin	3.29±1.65*	6.53±3.04*
^32^P-colloid+endostatin	2.142±1.09*^†^	4.36±1.89*^†^

### Pathological observation

**Figure 4:**
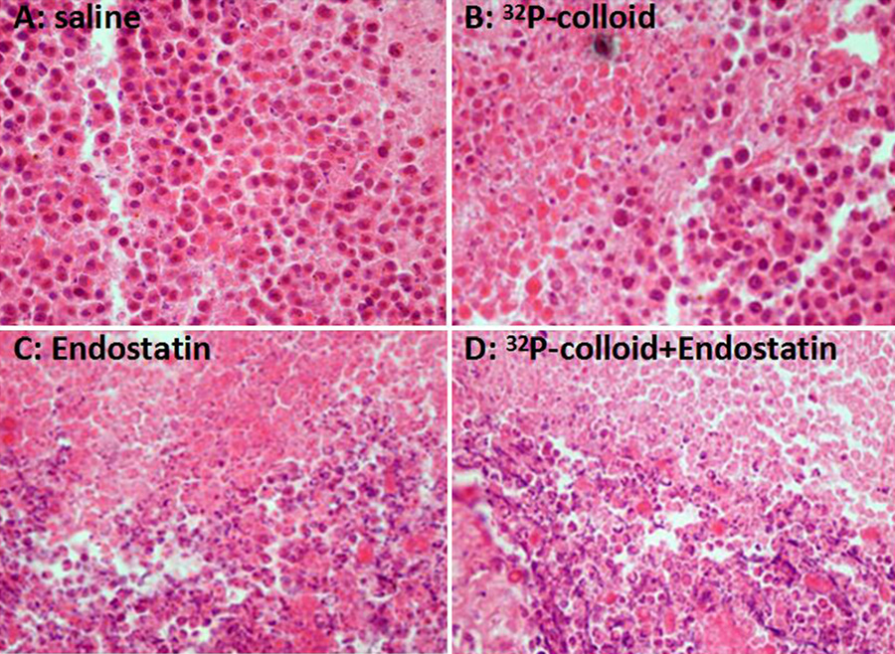
HE staining of tumour tissues

The tumour cells in the saline control group were closely arranged, exhibiting a few necrotic cells and a large quantity of neoplastic cells. Obvious necrosis and only few neoplastic cells were seen in the ^32^P-colloid and endostatin gene groups, whereas extensive necrosis and loosely arranged tumour cells were observed in the ^32^P-colloid combined with endostatin gene group (Figure 4).

**Figure 5:**
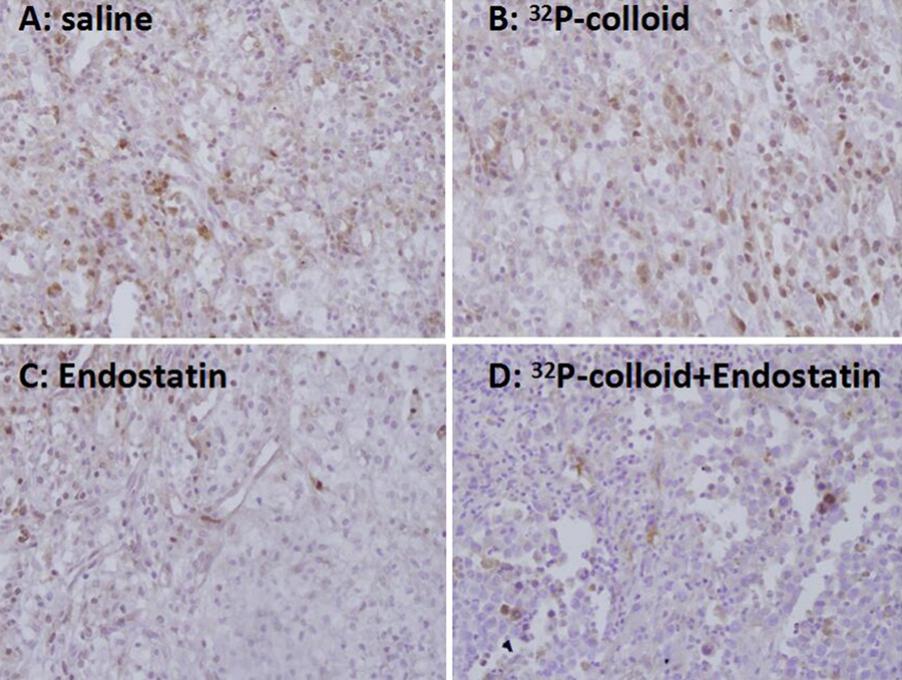
MVD measurement by immunohistochemistry

**Figure 6:**
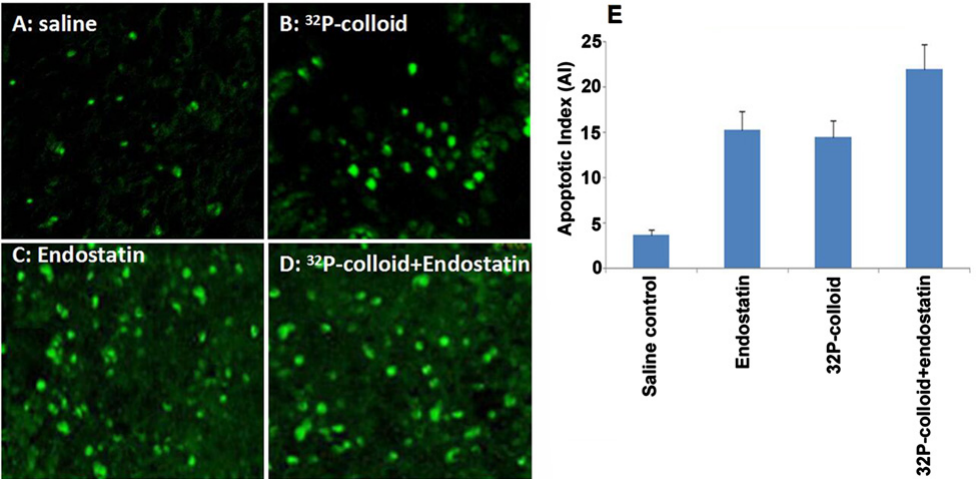
Apoptotic index measurement and quantification

### MVD measurement

A large amount of vascular endothelial cells were stained in the peripheral region of the tumour in the control saline group, whereas the number of stained endothelial cells in three treatment groups was significantly decreased (Figure 5). After the stained endothelial cells were counted, MVD was calculated as illustrated in Table 2. The MVD in all three treatment groups was significantly decreased as compared with that in the control saline group (*P*<0.001). The MVD in the ^32^P-colloid combined with endostatin group was the smallest, significantly differing from those in the ^32^P-colloid or endostatin gene alone group (*P*<0.05).

**Table 2:** Comparison of MVD count at 20 days after treatment among four groups (*x*±*S*) *P<0.001 compared with saline control group; ^†^*P*<0.05 compared with ^32^P-colloid group; ^‡^*P*<0.05 compared with ^32^P-colloid or endostatin group.

Group	MVD
Saline control	69.45±13.61
Endostatin	36.16±9.65*^†^
^32^P-colloid	41.72±11.20*
^32^P-colloid+endostatin	18.63±8.36*^‡^

### AI measurement

The representative imaging for AI measurement in each group was shown in Figures 6(A)–6(D). The differences in AI between all treatment groups and control group were statistically significant (*P*<0.001). No significant difference was observed between the ^32^P-colloid and endostatin gene groups (*P*>0.05), whereas the AI in the ^32^P-colloid combined with endostatin gene group was significantly higher than those in ^32^P-colloid and endostatin gene groups (both *P*<0.01) (Figure 6E).

## DISCUSSION

In the present study, the *in vivo* anti-tumour effect of ^32^P-colloids combined with endostatin was demonstrated. ^32^P is a pure beta-emitter, with an average length of 3-4 mm in soft tissues, 99% of the energy accumulated in the tumour tissue 4 mm adjacent to the injection site with a half-life of 14.28 days. Due to these characteristics, ^32^P has been widely used in internal radiation therapy for tumour treatment [10].

Several mechanisms have been proposed for the inhibitory effect of ^32^P colloid upon tumour growth [10]. Firstly, ^32^P can directly kill tumour cells. After injected into the tumours, ^32^P colloid is accumulated mainly in the tumour tissues due to the limited lymphatic drainage, resulting in much higher radioactivity within the tumour tissues than that in adjacent tissues. Subsequently, the tumour cells will be delivered with an absorbed lethal dose, whereas the adjacent tissues will be spared from irreversible radiation damage. In addition, because of relatively long half-life and retention in tumour tissues, ^32^P colloid can continuously irradiate tumour cells and lead to retention effect to overcome the radiation resistance acquired by DNA-damage repair. Secondly, ^32^P can induce tumour cell apoptosis, possibly through the DNA damage initiated by hydroxyl radicals. More importantly, internal radiation therapy rarely induces inflammation because of relative low dosage compared with the single high-dose external irradiation. This opinion could be supported by our results in the present study. Most rats in three treatment groups were in good condition and the tumour grew slowly without significant radiation damage or systemic symptoms. Thirdly, ^32^P is probably capable of inhibiting tumour angiogenesis. Compared with pure ionic colloidal solution, ^32^P has a larger particle diameter, which makes it easier to stay in the local neovascularization and cause lethal damage to peripheral vascular endothelial cells by continuous irradiation. In this way, the tumour regrowth and recurrence will be reduced because of disrupting the development of collateral circulation, which was also supported by the facts that the tumour growth rate and MVD in the ^32^P treatment were significantly lower compared with those in the control group.

Although ^32^P-colloid or endostatin alone could inhibit tumour growth, both therapies failed to completely prevent tumour growth, indicating the limitations of ^32^P-colloid or endostatin treatment alone. It has been reported that ^32^P-colloid inhibited tumour growth in a dose-dependent manner [10]. A higher dosage of ^32^P-colloid can kill more tumour cells, but also increases the risk of radiation injury. Endostatin targets the nascent endothelial cells rather than the tumour cells. Therefore, it can only indirectly inhibit tumour growth. In our study, endostatin was injected into the well-established tumour mass, in which most vascular endothelial cells were in a relative static state, had decreased proliferation ability and lacked sensitivity to endostatin. Bergers et al. [11] have demonstrated that the earlier the intervention time of endostatin was applied, the higher clinical efficacy was achieved. Tumour cells transfected with endostatin gene did not even form tumours after inoculation [12,13], suggesting that endostatin mainly targets the tumour tissues at advanced stage.

The tumour growth rate in the ^32^P-colloid combined with endostatin group was the lowest among all groups, even significantly lower than that in either ^32^P-colloid or endostatin alone group, suggesting that endostatin could enhance the effect of ^32^P-colloid on killing tumour cells, which was further supported by the massive tumour cell necrosis, sparsely arranged nascent tumour cells and significantly declined MVD in the ^32^P-colloid combined with endostatin group as compared with other groups. The results in this study were consistent with previous report [14,15].

The synergic effect of ^32^P-colloid combined with endostatin on killing tumour cells may be explained by the follow causes. First, it is more easily to transfect endostatin plasmid into the ^32^P-colloid-treated tumour cells. Second, the endothelial cells which are in static state and insensitive to endostatin could initiate the process of DNA damage repair after ^32^P-colloid irradiation and become sensitive to endostatin during this process. Third, in order to repair cell damage caused by ^32^P-colloid, a larger amount of angiogenesis is required but it could be inhibited by continuous expression of endostatin in our experiment.

Due to the side effects of anti-cancer medications constantly result in the failure of anti-tumour therapy, reducing the medication dosage under the premise to ensure the clinical efficacy is critical for successful treatment. Our study demonstrated that the combination of endostatin and ^32^P-colloid significantly enhances the inhibitory effects of ^32^P-colloid at a relatively low dosage on tumour growth, and thus reduces the radiation injury. In summary, ^32^P-colloid combined with endostatin achieved high *in vivo* inhibitory effects upon tumour growth, which may provide a novel strategy for tumour treatment.

## Abbreviations

AI: apoptotic index
MVD: microvessel density
